# Large animal model for retroperitoneal lymphatic and lung metastasis

**DOI:** 10.3892/mmr.2013.1720

**Published:** 2013-10-10

**Authors:** YONG-WEN HUANG, MING-FEI GUAN, JI-HONG LIU, CHUN-YAN LAN, TING WAN, XIN HUANG

**Affiliations:** 1 Department of Gynecology, State Key Laboratory of Oncology in South China, Sun Yat-sen University Cancer Center, Guangzhou, Guangdong 510060, P.R. China; 2 Department of Gynecology, Tumor Hospital of Shantou University Medical College, Shantou, Guangdong 515041, P.R. China

**Keywords:** retroperitoneal lymph nodes, lung metastasis, metastasis, VX2 carcinoma, rabbit model

## Abstract

Retroperitoneal lymph node and lung metastasis are important prognostic factors for gynecologic cancer. The present study aimed to develop a new animal model for retroperitoneal lymph node and lung metastasis. VX2 squamous cell carcinoma tumor tissues were injected into the left gastrocnemius muscle of 38 healthy female New Zealand white rabbits. Animals were randomized into three groups according to day of sacrifice: 1, day 19; 2, day 22; and 3, day 25. Implanted primary tumor (IPTu), left and right retroperitoneal lymph node volumes and lung wet weights were measured on the day of sacrifice. The IPTu and left and right retroperitoneal lymph node volumes increased in a time-dependent manner. In addition, the proportion of animals with metastasis to the left peritoneal lymph nodes and the number of nodes involved increased over time. For days 19, 22 and 25, the proportion of animals with nodal metastasis was 58.3, 84.6 and 100%, respectively, and the number of affected nodes (range) was 3 (2–3), 3 (3–5) and 4 (4–5), respectively. No metastasis was detected in the right peritoneal lymph nodes. Metastasis to the lungs also increased with time, but was not statistically significant at days 19, 22 and 25 with metastasis present in 33.3, 38.5 and 76.9% of animals, respectively. Rates of metastases to the left retroperitoneal lymph nodes and lungs were found to positively correlate with the volumes (r=0.416 and 0.449, respectively). The current study assessed the characterization of a rabbit VX2 carcinoma model. This animal model is likely to be useful for evaluating retroperitoneal lymph node and lung metastasis.

## Introduction

Retroperitoneal lymph nodes and lung metastases are an important route of dissemination and a significant prognostic factor for various types of gynecologic cancer, such as ovarian cancer (1,2). Retroperitoneal lymph node metastases are difficult to treat surgically since these lymph nodes are located close to the great vessels of the abdominopelvic cavity. Surgery becomes particularly difficult when the great vessels are involved. Current treatments for retroperitoneal lymphatic node and lung metastases include systemic chemotherapy, reduction in visible tumor burden and palliative therapy. Progress in the treatment of ovarian and other types of gynecologic cancer has been limited by difficulties in the removal of the retroperitoneal lymph nodes and the lack of studies focusing on suitable animal models for retroperitoneal lymphatic and lung metastasis.

Only a few experimental animal models of lung and lymph node metastasis have been developed that involve the use of mice, rats and rabbits (3–5). In rat and mouse models of lymph node metastases, cancer cells are injected directly into the arch cushion resulting in metastasis to inguinal lymph nodes (3,4). Fu and Hoffman (6) previously reported that human ovarian carcinoma metastatic models were constructed in nude mice by the orthotopic transplantation of histologically intact patient specimens. Due to limitations in body size, blood supply and tolerance to major surgical interventions and local chemotherapy, these models were suitable only for studies of treatment with systemic or intraperitoneal chemotherapy (3,4). Use of larger animals improves the ability to evaluate the efficacy of chemotherapy or other medically relevant procedures for treating metastatic cancer. Over the past two decades, rabbit animal models have been used to investigate lymph node metastasis (7–16), however, a number of these studies did not specifically characterize retroperitoneal lymph node metastasis. Characterization of metastasis to the retroperitoneal lymph nodes in a tumor-bearing rabbit have been previously reported only once and this involved injection of cancer cells into the endometrium (7).

The development of a large animal model of the metastasis of various types of gynecologic cancer to the retroperitoneal lymph nodes and lungs is highly desirable. Such models may be established by inoculation of the ovary or cervix with VX2 squamous cell carcinoma tissues. Rabbit VX2 carcinoma, which is a transplantable squamous cell carcinoma, was selected for establishment of this animal model since it is characterized by rapid growth and early metastasis. VXT rabbit models of cancer have previously been used to study types of renal, liver, lung, head and neck, brain and uterine cancer (7–9,16). The current study assessed the initial characterization of a rabbit VX2 carcinoma model designed to investigate metastasis to the retroperitoneal lymph nodes and lungs.

## Materials and methods

### Animals

In total, 41 female New Zealand white rabbits were obtained from the Animal Experimental Center of Sun Yat-sen University (Guangzhou, Guangdong, China) and kept in the Animal Biosafety Level 3 Laboratory of the Animal Experimental Center of Sun Yat-sen University (Animal Study Certificate 0027164). One animal (the carrier) had previously been implanted with VX2 cells and the other animals were healthy. Rabbits were 8–9-weeks-old and had body weights of 1.7–2.2 kg (median, 1.85 kg). The animals were individually housed, allowed free access to standard laboratory food and water and were subjected to daily 12-h light/dark cycles. The animal protocol used was approved by the Animal Welfare Committee of the State Key Laboratory of Oncology in South China.

### Preparation of the animal model

Construction of the retroperitoneal lymph node animal model required two transplantation steps. First, it was necessary to transfer tumor cells from the carrier rabbit to a healthy rabbit (referred to as the transfer animal) to ensure that the VX2 tumor cells, which were to be used in the model system, had optimal viability. Secondly, tumor tissue from the transfer animal was transplanted into the experimental rabbits (n=39).

To generate the transfer animal, the carrier animal was anesthetized by injection of ketamine (10 mg/kg) into the ear vein. The volume of the tumor in the carrier animal was ~283.5 cm^3^ at 45 days post-transplantation. Following the preparation and disinfection of the skin in the area of the tumor, fresh tumor tissue was excised, flushed with 0.9% sodium chloride solution, and placed in RPMI 1640 medium (Gibco, Life Technologies, Carlsbad, CA, USA). Excised tumor tissue was minced into fragments of about 0.5 to 1.0 mm3, suspended in RPMI 1640 medium (Gibco). Following suspension, the tumor cell concentration was adjusted to 20% (~1×10^10^ cells/ml), drawn into a 2 ml lumbar puncture needle and injected into the left gastrocnemius of a healthy rabbit that served as the transferring animal. On post-inoculation day 30, the transfer rabbit was sacrificed by aeroembolism (injection of air into the ear vein) and the tumor was removed for analysis and construction of the retroperitoneal lymph node animal model.

To generate the animal model, the tumor from the transfer animal was excised, suspended and injected into the 39 healthy rabbits as described above. The rabbits bearing VX2 carcinoma cells were randomly divided into 3 groups according to the day the rabbits were sacrificed following transplantation: 1, day 19; 2, day 22; and 3, day 25.

Following surgery, the animals were observed daily for food intake, activity and the presence of abnormalities, such as diarrhea or dehydration. Implanted primary tumor (IPTu) size was measured every 2 days following post-transplantation. Animals were euthanized by aeroembolism and post-mortem examinations included determination of gross tumor pathology, tumor size, tumor distribution, and local morphological features, which were performed by YW Huang to avoid differing judgments. The volume of the IPTu and left and right retroperitoneal lymph nodes, as well as the wet weights of the lungs were measured. The volume of the lymph node was calculated using the formula: Volume = length × (width^2^)/2

Critical tissues, including IPTu, left and right retroperitoneal lymph nodes and lungs, were stained with hematoxylin and eosin to evaluate tissue histopathology. For swollen lymph nodes observable by the naked eye, as well as visible pulmonary metastases, 3 serially sectioned, 4-μM samples were evaluated per sample. For lymph nodes without enlargement as observed by the naked eye, a total of five sections (4 μM) including the 5th, 10th, 15th, 20th, and 25th sections from 25 serial sections were selected for analysis. Similarly, for lung tissue without metastases as observed by the naked eye, a total of five sections (4 μM), including the 10th, 20th, 30th, 40th and 50th sections from a total 50 serial sections, were chosen for analysis. The slides were evaluated by two pathologists who were blinded to the experiment. The rates of metastases were calculated as per rabbit with at least one positively identified metastatic tumor. The rates of metastases to the lymph nodes and lungs, positive lymph node number and lung wet weights were compared among the 3 groups.

### Statistical analysis

Comparability among the 3 groups was tested using one-way analysis of variance (ANOVA) for continuous variables and χ^2^/Fisher's exact test for categorical variables. The differences among the groups for the number of positive left retroperitoneal lymph nodes was determined by Kruskal-Wallis one-way analysis of variance. When a significant difference among the groups was detected, multiple comparison tests were performed using the Bonferroni method with type-I error adjustment. The difference between the left and right retroperitoneal lymph nodes within each group was tested using the paired t-test. Continuous variables were presented as mean ± SD and categorical data were represented by number (n) and percentage (%). In addition, the number of positive left retroperitoneal lymph nodes was presented as the median (range). The point biserial correlation coefficient was used to test the correlation between a continuous and a categorical variable with two levels. All statistical assessments were two-sided and P<0.05 was considered to indicate a statistically significant difference. Statistical analyses were performed using SPSS 15.0 statistics software (SPSS Inc., Chicago, IL, USA).

## Results

### Survival, implantation and growth of the primary tumor

**Figure 1:**
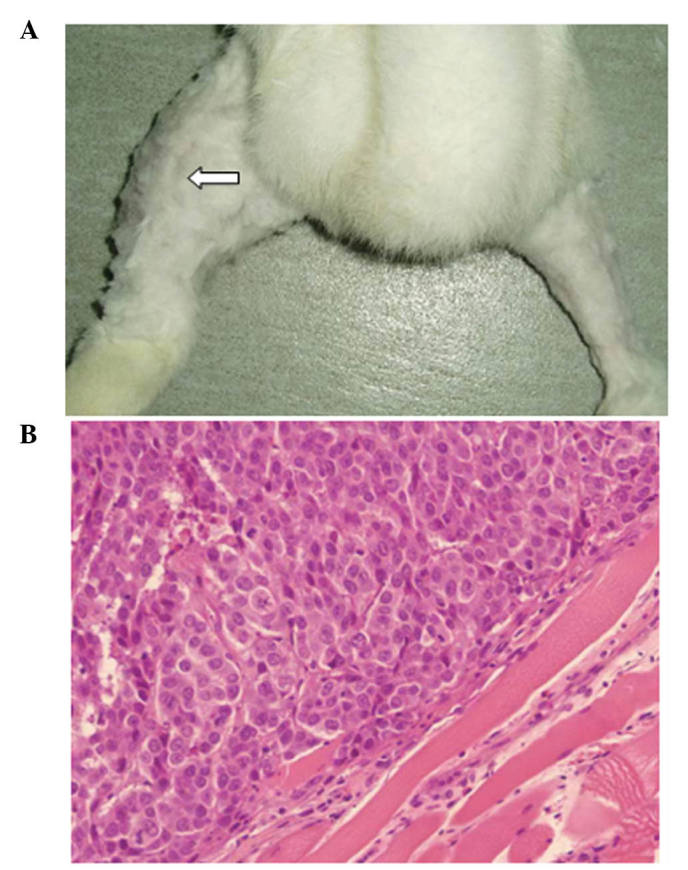
(A) Presence of a primary tumor, indicated by the arrow and (B) metastatic squamous carcinoma cells (magnification, 20×10) in the left gastrocnemius muscle of a rabbit 19 days following inoculation of the gastrocnemius muscle with VX2 cells.

Of the 39 animals that were transplanted with VX2 tumor cells, 38 survived prior to sacrifice; one rabbit in group 1 died of diarrhea of unknown cause on day 5. In all the surviving animals, a primary tumor was determined in the injected gastrocnemius muscle (Fig. 1A and B).

A significant difference was identified in the volume of the IPTu among the 3 groups (all P<0.001; Table I). In addition, a significant difference was identified in the volume of the IPTu between groups 1 and 3 (P<0.001) and groups 1 and 2 (P=0.041).

### Metastasis of retroperitoneal lymph nodes

**Figure 2:**
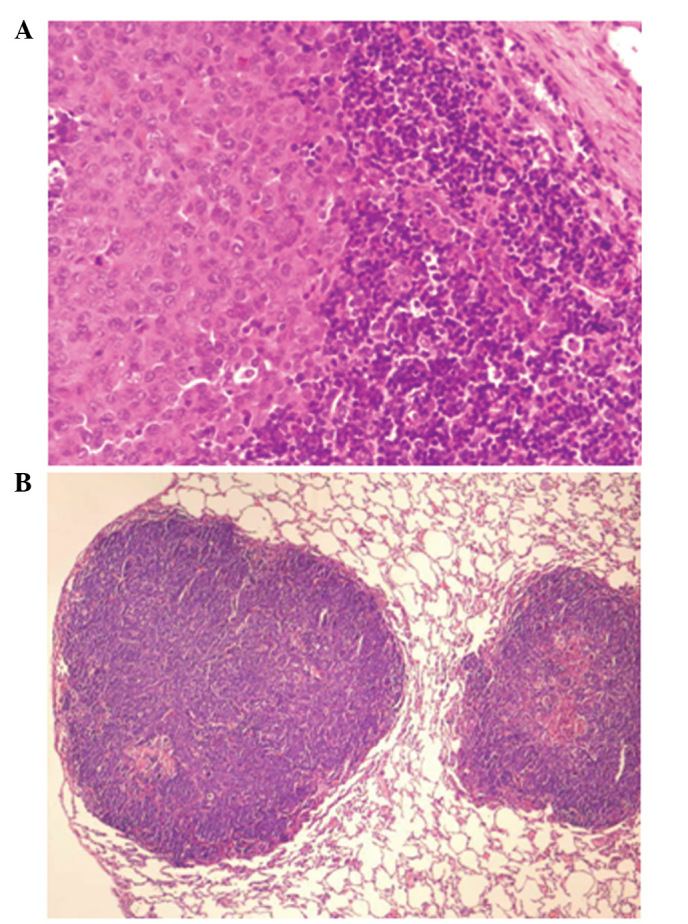
(A) Metastatic squamous carcinoma cells in the left retroperitoneal lymph nodes (magnification, 20×10) and (B) microscopic appearance of metastatic carcinoma present in the lungs (magnification, 4×10) of a rabbit 25 days following inoculation of the gastrocnemius muscle with VX2 cells.

Metastasis was detected in the left retroperitoneal lymph nodes (Fig. 2A). For these lymph nodes the volume, rate of metastasis and number of positive lymph nodes were found to be significantly different among the 3 groups (all P≤0.05; Table I). For lymph node volume, group 3 was significantly different compared with groups 1 and 2 (both P<0.001; Table I) while the volumes of groups 1 and 2 remained similar (P=1.00). A significant difference was identified in the rates of metastasis (percentage of animals with metastasis/day of sacrifice) to the left retroperitoneal lymph nodes among the three groups (P=0.019). This significant difference reflected differences between groups 1 and 3 (P=0.015), however, the rate was found to be similar between groups 1 and 2 and groups 2 and 3 (P=0.202 and P=0.480, respectively). The differences among the groups for the number of positive left retroperitoneal lymph nodes resulted from a significant difference between groups 1 and 3 (P=0.001) and groups 2 and 3 (P<0.001). In addition, the number of positive lymph nodes was found to be significantly different between groups 1 and 2 (P=0.029). The rate of metastasis to the left peritoneal lymph node was found to positively correlate with lymph node volume (r=0.416; P=0.009; data not shown).

**Figure 3:**
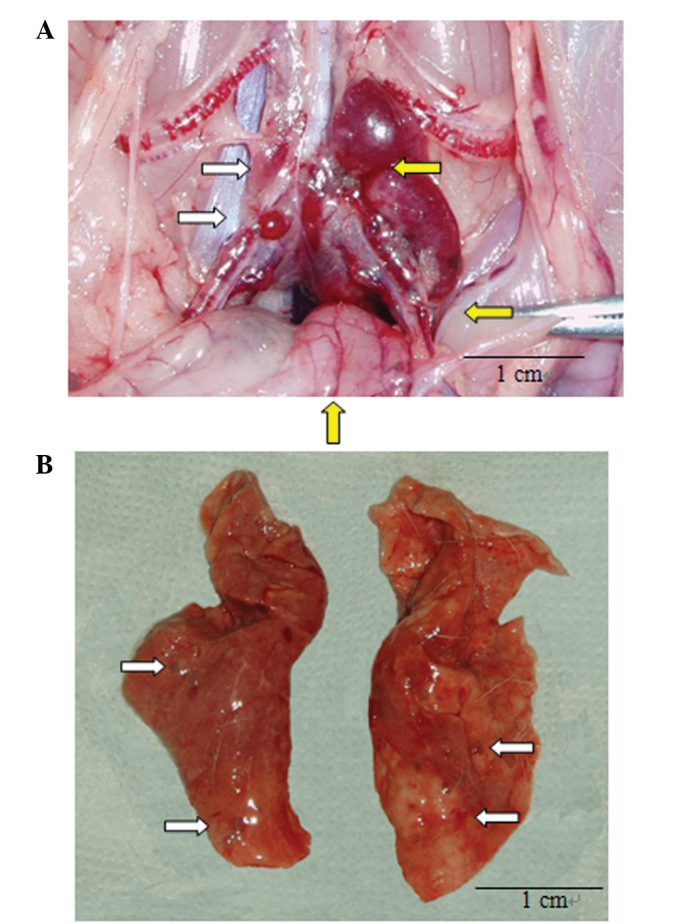
Macroscopic appearances of the (A) left (yellow arrows) and right (white arrows) retroperitoneal lymph nodes and (B) bilateral lungs (white arrows indicate metastatic carcinoma) of a rabbit 25 days following inoculation of the gastrocnemius muscle with VX2 cells.

In contrast to the left retroperitoneal lymph nodes, no metastasis was detected in the right retroperitoneal lymph nodes, however, the volume of these lymph nodes differed among the 3 groups (P<0.001; Table I). Significant differences were identified in the volumes between groups 1 and 3 and groups 2 and 3 (both P<0.017), but lymph node volumes were similar between groups 1 and 2 (P=1.000). For all 3 groups, on the day of sacrifice, the volume of the right peritoneal lymph node was considerably smaller than that of the left (all P<0.001; Fig. 3A).

### Metastasis of the lungs

For the animals sacrificed on post-VX2 cell inoculation day 25, macroscopic metastatic lesions were visible at the edge of the bilateral lungs (Fig. 3B) and microscopic and macroscopic appearances of metastatic carcinoma were present (Figs. 2B and 3B). Slight differences were found in the rates of metastases to the lung and wet weights of the lungs among the 3 groups, but were not found to be statistically significant (P=0.055; Table I). However, a significantly positive correlation was found between the rate of metastasis to the lung and the wet weight of the lung (r=0.449; P=0.005).

## Discussion

The present study has focused on the characterization of a VX2 rabbit model for the metastasis of squamous cell carcinoma to the retroperitoneal lymph nodes and lungs. The pattern of metastasis of the primary tumor from the left gastrocnemius to the left retroperitoneal lymph nodes and lungs is comparable with that of patients with metastasizing types of gynecologic cancer. By days 19, 22 and 25 following inoculation, the percentages of animals which had developed primary tumors in the gastrocnemius muscle were 92.3, 100 and 100%, respectively. The proportion of animals with metastasis to the lungs or retroperitoneal lymph nodes increased over the duration of the study. For example, at days 19, 22 and 25, it was found that 58.3, 84.6 and 100% of the animals exhibited metastasis to the left retroperitoneal lymph node, respectively and 33.3, 38.5 and 76.9% exhibited lung metastasis, respectively. Similarly, the wet weight of the lungs and volume of left retroperitoneal lymph nodes increased with time, consistent with increased mass due to tumor metastasis. In addition, the number of involved left retroperitoneal lymph nodes increased in a time-dependent manner. The rate of metastasis (number of animals with metastasis/day of sacrifice) to the left retroperitoneal lymph node was found to positively correlate with lymph node volume and wet lung weight (r=0.416 and r=0.449, respectively). No metastasis was detected for the duration of the 25-day study to the right retroperitoneal lymph nodes and increase in the volume in this tissue was reduced compared with that of the left retroperitoneal lymph nodes.

Since only approximately 77% of animals exhibited lung metastasis at 25 days post-inoculation, an increased study duration may be required to investigate lung metastasis in this model system. The time-dependent increase in metastasis is likely to reflect the natural progression of metastasis in this system. Additional experiments are required to improve the characterization of this system in order to gain insight into how changes in the primary tumor and metastasis reflect the development of advanced cancer in humans.

Lymph node volume was used in the present study as an indication of metastasis based on the evidence that in the clinical visual detection of enlarged lymph nodes, radiographic images are an important indication of the possible metastasis. However, further microscopic analysis is required for a positive diagnosis since, as detected in the current study, enlarged lymph nodes are not always indicative of metastasis. In the current study, it was assumed for the lungs that the presence of a tumor is likely to result in an increase in wet lung weight and a correlation was identified between wet lung weight and metastasis.

During the current study it was found that the left popliteal fossa exhibited an enlarged lymph node 12 days post-inoculation and the proportion of rabbits with metastasis to this lymph node increased with time. At days 19, 22 and 25 the percentage of animals with left popliteal fossa metastasis was 16.67, 38.46 and 61.54%, respectively (P=0.072; χ^2^ test). It is unclear why the rate of metastasis to the left retroperitoneal lymph node was greater compared with that of the left popliteal lymph nodes. To address this issue, future studies are required to investigate the route of metastasis to these tissues using lymphangiography and other technologies.

The location of transplantation may affect the site of metastasis. Transplantation of VX2 tumor tissue into the pyriform sinus submucosa of rabbits resulted in deep cervical lymph node metastasis at 14 days post-transplantation (17). However, rates of submandibular lymph node metastasis were found to be 60, 80 and 100% at 14, 21 and 28 days post-inoculation, respectively (17). Additionally, rates of paratracheal lymph node metastasis were reported to be 0, 80 and 100% at 14, 21 and 28 days following inoculation, respectively (17). Mechanisms responsible for the observed higher rates of metastases to the deep cervical lymph nodes compared with local lymph nodes adjacent to orthotopic tumors remain to be identified.

The VX2 tumor is a transplantable rabbit squamous carcinoma characterized by rapid growth, stable physiological characteristics and early metastasis (18). VX2 cells were selected primarily, since they are the only existing cells to induce squamous cell carcinoma in rabbits. It is known that adenocarcinoma is the most common type of endometrial cancer (EC), however, squamous cell carcinoma is observed in patients with partial EC. Furthermore, squamous cell carcinoma and adenocarcinoma are epithelial tumors and the biological characteristics of their retroperitoneal lymph node metastasis are similar. Although potentially different from adenocarcinoma, this squamous cell carcinoma is extremely malignant and may be implanted at almost any site in rabbits. A primary tumor model involving VX2 may be constructed following transplantation into rabbit liver, kidney, lung, breast or uterus (8,9,16,19,20). VX2 rabbit models of cancer have previously been used to investigate lymph node metastasis (5,7,11–15). However, the majority of these studies were imaging studies that did not specifically characterize retroperitoneal lymph node metastasis. One previous study characterized a VX2 rabbit model of retroperitoneal lymphatic metastasis (7). Results of that study differed from those of the present study as the VX2 tumor grafts were established by orthotopic embedding of the VX2 cells into the endometrium. The study found that 100% of the animals developed tumors and metastasis to the retroperitoneal lymph nodes which occurred within 1 and 3 weeks. The rate of metastasis to the retroperitoneal lymph nodes was similar to the results of the current study, however, the endometrium-based model has highlighted inconsistent results (unpublished data). Additional analyses with regard to the effect of the location of the primary tumor on metastasis to the retroperitoneal lymph nodes are required to establish the best VX2 rabbit model.

Metastases to the retroperitoneal lymph nodes and lungs are a serious challenge to clinicians who treat various types of gynecologic cancer. In patients with these types of cancer, retroperitoneal metastasis is present in ≥22% of cases (21,22). For the majority of types of gynecologic cancer, retroperitoneal metastasis is a characteristic of the International Federation of Gynecology and Obstetrics stage III and IV cancer classification and is an important prognostic factor (1,2,23,24). In patients with advanced stages of gynecologic cancer, control of lung metastasis is also essential for patient quality of life and increased survival rates (25).

Current treatments for retroperitoneal lymphatic and lung metastases include systemic chemotherapy, reduction in visible tumor burden and palliative therapy. However, results have indicated that the treatment of nodal metastasis with chemotherapy may not control the disease (26). One study has previously reported that in ovarian cancer patients, retroperitoneal lymph node involvement was present in 35% (35/100) of those treated with surgery and 54% (15/28) and 36% (28/77) of those who also received 3 or 6 courses of chemotherapy, respectively (26). These observations indicate that new treatment regimens for these types of cancer are required.

One limitation of the present study was that a large majority of ovarian carcinomas are adenocarcinomas, not squamous carcinomas. Thus, the manner in which this VX2 model reflects the biology of adenocarcinoma-derived metastases in various types of gynecologic cancer is unclear. Additional studies are required to understand how this rabbit model reflects the metastasis of gynecologic cancer in humans.

In conclusion, the present study determined a unique rabbit model of the metastasis of squamous carcinoma cells to the retroperitoneal lymph nodes and lungs. This model is likely to be useful in understanding how various types of cancer (regardless of the primary tumor site) metastasize to the retroperitoneal lymph nodes and lungs.

## Figures and Tables

**Table I:** IPTu, LRLN and RRLN volumes among the 3 groups.

Variable	Group 1 (n=12)	Group 2 (n=13)	Group 3 (n=13)	P-value
Mean ± SD IPTu volume, cm^3^	24.62±4.97...	31.64±5.52..	57.65±8.95^f,g^	<0.001^a,h^
Mean ± SD LRLN volumes, cm^3^	0.54±0.12.	0.65±0.15	1.84±0.47^f,g^	<0.001^a,h^
Mean ± SD RRLN volumes, cm^3^	0.011±0.004^e^	0.012±0.009^e^	0.020±0.007^e–g^	<0.001^a,h^
Metastases to LRLN, n (%)	7 (58.3)	11 (84.6)	13 (100)^f^	0.019^b,h^
Median positive LRLN (range)	3 (2–3)	3 (3–5)	4 (4–5)^f,g^	<0.001^c,h^
Metastases to the lung, n (%)	4 (33.3)	5 (38.5)	10 (76.9)	0.055^d^
Mean ± SD lung weight, g	8.52±0.65	8.91±1.10	9.04±0.76	0.302^a^

P-values were determined by ^a^ANOVA, ^b^Fisher's exact test, ^c^Kruskal-Wallis one-way analysis of variance and ^d^χ^2^ test. P<0.05 was considered to indicate a statistically significant difference between ^e^LRLN and RRLN using paired t-test; ^f^groups 3 and 1; ^g^groups 2 and 3; and ^h^among the 3 groups. Pairwise multiple comparisons between groups were determined using Bonferroni's test with α=0.017 adjustment. IPTu, implanted primary tumor; LRLN, left retroperitoneal lymph nodes; RRLN, right retroperitoneal lymph nodes; ANOVA, analysis of variance.

## Abbreviations

IPTu: implanted primary tumor

## References

[1] Pecorelli S, Benedet JL, Creasman WT, Shepherd JH, International Federation of Gynecology and Obstetrics (1999). FIGO staging of gynecologic cancer. 1994–1997 FIGO Committee on Gynecologic Oncology. Int J Gynaecol Obstet.

[2] Kanazawa K, Suzuki T, Tokashiki M (1999). The validity and significance of substage IIIC by node involvement in epithelial ovarian cancer: impact of nodal metastasis on patient survival. Gynecol Oncol.

[3] Szekeres T, Saiko P, Fritzer-Szekeres M, Djavan B, Jäger W (2011). Chemopreventive effects of resveratrol and resveratrol derivatives. Ann NY Acad Sci.

[4] Trencsenyi G, Kertai P, Bako F (2009). Renal capsule-parathymic lymph node complex: a new in vivo metastatic model in rats. Anticancer Res.

[5] Servais EL, Colovos C, Bograd AJ, White J, Sadelain M, Adusumilli PS (2011). Animal models and molecular imaging tools to investigate lymph node metastases. J Mol Med (Berl).

[6] Fu X, Hoffman RM (1993). Human ovarian carcinoma metastatic models constructed in nude mice by orthotopic transplantation of histologically-intact patient specimens. Anticancer Res.

[7] Chang S, Gu M, Wu F (2000). Establishment of transplanted endometrial neoplasm model in rabbit and its biological features. Prog Obstet Gynecol.

[8] Harima Y, Harima K, Hasegawa T, Shikata N, Tanaka Y (1996). Histopathological changes in rabbit uterus carcinoma after transcatheter arterial embolization using cisplatin. Cancer Chemother Pharmacol.

[9] Harima Y, Harima K, Hasegawa T, Shikata N, Tanaka Y (1996). Transcatheter arterial embolization with cisplatin: apoptosis in VX2 tumour uterus transplants. Anticancer Res.

[10] Ishibashi S, Sonoda K, Fujii K, Ishikawa K, Shiraishi N, Kitano S (2004). A convenient murine model for the study of intra-abdominal lymph node metastasis. Oncol Rep.

[11] Choi SH, Kim KH, Moon WK, Kim HC, Cha JH, Paik JH, Chang KH (2010). Comparison of lymph node metastases assessment with the use of USPIO-enhanced MR imaging at 1.5 T versus 3.0 T in a rabbit model. J Magn Reson Imaging.

[12] Choi SH, Moon WK, Hong JH (2007). Lymph node metastasis: ultrasmall superparamagnetic iron oxide-enhanced MR imaging versus PET/CT in a rabbit model. Radiology.

[13] Herborn CU, Lauenstein TC, Vogt FM, Lauffer RB, Debatin JF, Ruehm SG (2002). Interstitial MR lymphography with MS-325: characterization of normal and tumor-invaded lymph nodes in a rabbit model. AJR Am J Roentgenol.

[14] Wang L, Yao Q, Wang J (2008). MRI and hybrid PET/CT for monitoring tumour metastasis in a metastatic breast cancer model in rabbit. Nucl Med Commun.

[15] Tsuda N, Tsuji T, Kato N (2005). Interstitial magnetic resonance lymphography using gadolinium-ethoxybenzyl-diethylenetriamine pentaacetic acid in rabbits with lymph node metastasis. Invest Radiol.

[16] Rhee TK, Ryu RK, Bangash AK (2007). Rabbit VX2 tumors as an animal model of uterine fibroids and for uterine artery embolization. J Vasc Interv Radiol.

[17] Shen N, Wu H, Xu X, Wang J, Hoffman MR, Rieves AL, Zhou L (2009). Cervical lymph node metastasis model of pyriform sinus carcinoma. ORL J Otorhinolaryngol Relat Spec.

[18] Kidd JG, Rous P (1940). A transplantable rabbit carcinoma originating in a virus induced papilloma and containing the virus in masked or altered form. J Exp Med.

[19] Horkan C, Ahmed M, Liu Z, Gazelle GS, Solazzo SA, Kruskal JB, Goldberg SN (2004). Radiofrequency ablation: effect of pharmacologic modulation of hepatic and renal blood flow on coagulation diameter in a VX2 tumor model. J Vasc Interv Radiol.

[20] Chen JH, Li Y, Yao Q, Ling R, Wang L, Li KZ, Wang Z, Chen T (2005). Treatment of rabbits bearing advanced VX2 tumors in the mammary gland with nano-sized liposomal adriamyc in administered by various routes. Natl Med J China.

[21] Maggioni A, Benedetti Panici P, Dell'Anna T (2006). Randomised study of systematic lymphadenectomy in patients with epithelial ovarian cancer macroscopically confined to the pelvis. Br J Cancer.

[22] Angioli R, Plotti F, Palaia I (2008). Update on lymphadenectomy in early and advanced ovarian cancer. Curr Opin Obstet Gynecol.

[23] Bolger BS, Dabbas M, Lopes A, Monaghan JM (1997). Prognostic value of preoperative squamous cell carcinoma antigen level in patients surgically treated for cervical carcinoma. Gynecol Oncol.

[24] Shigematsu N, Ito H, Nishiguchi I (1997). Prognostic factors of cervical carcinoma treated with postoperative radiotherapy. Nippon Igaku Hoshasen Gakkai Zasshi.

[25] Peiretti M, Zapardiel I, Zanagnolo V, Landoni F, Morrow CP, Maggioni A (2012). Management of recurrent cervical cancer: a review of the literature. Surg Oncol.

[26] Morice P, Joulie F, Rey A (2004). Are nodal metastases in ovarian cancer chemoresistant lesions? Analysis of nodal involvement in 105 patients treated with preoperative chemotherapy. Eur J Gynaecol Oncol.

